# Low-Molecular-Weight Fucoidan from *Undaria pinnatifida* Mitigates *Salmonella*-Induced Injury Through Gut Microbiota and Immune Regulation

**DOI:** 10.3390/foods15122135

**Published:** 2026-06-13

**Authors:** Lu Wang, Zhixiu Xiao, Jiaxin Yang, Chunyan Lu, Xiaomeng Ren, Shuang Song, Jinchi Jiang, Chunqing Ai

**Affiliations:** 1 State Key Laboratory of Marine Food Processing & Safety Control, School of Food Science and Technology, Dalian Polytechnic University, Dalian 116034, China; 13624080614@163.com (L.W.); yang18342388117@163.com (J.Y.); rxmfighting@163.com (X.R.); songs1008@163.com (S.S.); jiangjinchi@njtech.edu.cn (J.J.); 2 Department of Oncology, Qingdao Municipal Hospital, Qingdao 266071, China; xiaozx67@163.com (Z.X.); knfengkai@163.com (C.L.)

**Keywords:** *Salmonella*, macrophage, gut microbiota, fucoidan

## Abstract

*Salmonella* primarily affects the gastrointestinal tract, causing local and systemic symptoms. Fucoidan exhibits therapeutic potential against *Salmonella*-induced pathology; however, the influence of its molecular weight on efficacy remains poorly understood. In this study, low-molecular-weight fucoidan from *Undaria pinnatifida* (LUPF) was prepared and characterized, and its protective effects against *Salmonella* infection were evaluated in a mouse model. LUPF effectively mitigated *Salmonella*-induced multiple organ damage by preserving mucin secretion and tight junction protein expression. Metabolomics analysis further demonstrated that LUPF normalized *Salmonella*-induced metabolic disturbances, thereby reducing systemic dysfunction. Mechanistically, LUPF suppressed inflammation by inhibiting mitogen-activated protein kinase (MAPK) and nuclear factor-κB (NF-κB) signaling pathways, while alleviating oxidative stress through activation of the Nrf2 pathway. In addition, LUPF restored gut microbiota homeostasis by reducing Proteobacteria levels, improving the Bacteroidota/Firmicutes ratio, enriching beneficial taxa, and enhancing short-chain fatty acid production. In vitro experiments further revealed that LUPF attenuated *Salmonella*-induced inflammation by modulating macrophage polarization. Collectively, these results suggest that LUPF has promising potential as a prebiotic candidate for reducing the risk of *Salmonella*-associated diseases.

## 1. Introduction

*Salmonella* enterica is a Gram-negative bacterium responsible for approximately 1.3 billion cases of illness annually [1]. More than 2500 serotypes have been identified, causing a wide range of clinical manifestations, from mild diarrhea to severe bacteremia, depending largely on the infecting serotype and host immune status [2]. Among them, *S. enterica* serovar Typhimurium (ST) is one of the major causes of foodborne gastrointestinal (GI) infection and is commonly transmitted through contaminated food, water, or contact with infected individuals [3]. Although antibiotics remain the standard treatment for salmonellosis, their widespread use is increasingly restricted due to gut dysbiosis, immune suppression, and the emergence of multidrug-resistant strains [4]. These challenges highlight the urgent need for safe and effective alternative strategies for the prevention and management of *Salmonella* infection.

Gut microbiota plays a critical role in defending against enteric pathogens through mechanisms including competitive exclusion, immune modulation, and the production of beneficial metabolites [5]. Consequently, microbiota-targeted strategies have attracted increasing research interest. Among these, polysaccharides derived from natural sources have emerged as promising candidates for mitigating pathogen-induced injury due to their ability to modulate gut microbial composition and function [6]. Fucoidan, a sulfated polysaccharide derived from brown seaweeds and characterized by fucose as its signature monosaccharide, exhibits immunomodulatory, antioxidant, and anti-inflammatory properties [7,8,9], many of which are closely associated with its capacity to modulate the gut microbiota and metabolite profiles. Natural *Undaria pinnatifida* fucoidan (UPF, >290 kDa), derived from this widely consumed brown seaweed, has been reported to reduce ST-induced mortality and alleviate gut microbiota dysbiosis in mice [10,11]. These results suggest that fucoidan has great potential as a promising prebiotic candidate for alleviating diseases induced by pathogens.

However, the practical application of natural fucoidan is limited by its high molecular weight (Mw), which may result in low absorption and limited bioavailability. In contrast, low-Mw fucoidan generally exhibits enhanced antioxidant and anti-inflammatory bioactivities, along with improved biological utilization [12,13]. A comprehensive review indicates that low- to medium-Mw and highly sulfated fractions of UPF exhibit enhanced biological activities, highlighting the importance of structural modifications for improving their therapeutic potential [14]. For example, low-Mw fucoidan from *Sargassum crassifolium* (5–30 kDa) exhibits superior antibacterial activity against *Escherichia coli* in vitro, suggesting improved bioavailability [15]. Previous studies have shown that low-Mw fucoidan (<10 kDa) exerts stronger anti-inflammatory activity than natural fucoidan in LPS-stimulated inflammatory cell models by suppressing pro-inflammatory mediators and regulating macrophage responses [16,17]. However, these findings are mainly derived from in vitro models, while in vivo evidence regarding its protective effects against enteric pathogen infection, such as ST infection, remains limited. Therefore, as a logical continuation of our previous work [11], the present study prepared low-Mw fucoidan (LUPF) and evaluated its protective effects against ST infection in an in vivo model.

In this study, LUPF was prepared by acid hydrolysis and structurally characterized using multiple analytical methods. The protective effects of LUPF against ST-induced intestinal and systemic injury were evaluated in a mouse model, with a focus on intestinal barrier integrity, inflammatory signaling pathways, oxidative stress responses, gut microbiota composition, and serum metabolic profiles. In addition, the direct anti-inflammatory effects of LUPF on ST-induced inflammation were further assessed using an in vitro model. This study aims to provide a more comprehensive understanding of the protective mechanisms of low-Mw fucoidan and to evaluate its potential as a prebiotic candidate for reducing the risk of *Salmonella*-associated diseases.

## 2. Materials and Methods

### 2.1. Salmonella Strains and Chemical Reagents

*S. Typhimurium* ATCC14028 was preserved in our laboratory. Gentamicin sulfate (G6064) and cefradine (C804452) were purchased from Macklin Biochemical Technology Co., Ltd. (Shanghai, China). Antibodies against extracellular signal-regulated kinases (ERKs, ab184699), phosphorylated ERKs (p-ERKs, ab201015), c-Jun N-terminal kinases (JNKs, ab179461), phosphorylated JNKs (p-JNKs, ab124956), nuclear factor erythroid 2-related factor 2 (Nrf2, ab62352), inhibitor of nuclear factor-κB alpha (IκBα, ab76429), mucin2 (MUC2, ab272692), ZO-1 (ab276131), claudin (ab214487), and occludin (ab216327) were obtained from Abcam (Cambridge, MA, USA). Antibodies against p38 (4511), phosphorylated p38 (p-p38, 8960), NF-κB p65 subunit (p65, 8242T), and β-actin (4970T) were purchased from Cell Signaling Technology, Inc. (Danvers, MA, USA). Thiazolyl blue tetrazolium bromide (MTT, M8180) and Dimethyl Sulfoxide (DMSO, D8371) were supplied by Beijing Solarbio Science & Technology Co., Ltd. (Beijing, China). Nitric oxide (NO) assay kit was purchased from Beyotime Biotech Inc. (Shanghai, China).

### 2.2. Preparation of LUPF

Natural UPF was purchased from Qingdao Bright Moon Seaweed Group Co., Ltd. (Qingdao, China). According to our previous study [11], the UPF had a Mw of ~290 kDa, with galactose (Gal) and fucose (Fuc) as the major monosaccharides (Gal/Fuc molar ratio 1.35). Based on preliminary optimization experiments, UPF was hydrolyzed with 0.3 mol/L HCl at 90 °C for 30 min, followed by neutralization to pH 7.0. The hydrolysate was sequentially dialyzed using 4.5 kDa and 1 kDa Mw cut-off dialysis bags to remove high- and low-Mw fractions, respectively. The obtained LUPF fraction was further purified using a Bestdex G-25 gel filtration column and lyophilized by vacuum freeze-drying. The yield of purified LUPF relative to the starting UPF was approximately 20% (*w*/*w*).

### 2.3. Structural Characterization of LUPF

#### 2.3.1. Chemical Composition of LUPF

Total carbohydrate content was quantified using the phenol–sulfuric acid method [18]. Protein content was determined using a BCA assay kit (Beijing Solarbio Science & Technology Co., Ltd., Beijing, China). Sulfate content was measured by ion chromatography [19]. Uronic acid content was determined using the meta-hydroxybiphenyl method [20].

#### 2.3.2. Monosaccharide Composition

The monosaccharide composition of LUPF was analyzed by high-performance liquid chromatography (HPLC) following pre-column derivatization with 1-phenyl-3-methyl-5-pyrazolone, and monosaccharides were identified by comparing their retention times with authentic standards [21].

#### 2.3.3. Determination of Mw

The Mw of LUPF was determined by high-performance gel permeation chromatography (HPGPC) using an HPLC system (Waters; Milford, MA, USA) [22].

#### 2.3.4. Desulfation of LUPF

Desulfation of LUPF was performed according to a previously established method [23]. Briefly, 200 mg of LUPF was dialyzed against 0.1 M pyridine hydrochloride and lyophilized, followed by reaction in dimethyl sulfoxide/methanol/pyridine mixture (87:10:3, *v*/*v*/*v*) at 100 °C for 6 h. The reaction mixture was subsequently dialyzed against distilled water and lyophilized to obtain the desulfated product (ds-LUPF).

#### 2.3.5. Fourier-Transform Infrared Spectroscopy (FT-IR)

FT-IR spectra of LUPF were recorded using a PerkinElmer spectrometer (Waltham, MA, USA) over the range of 4000–400 cm^−1^.

#### 2.3.6. Nuclear Magnetic Resonance (NMR) Spectroscopy

In total, 30 mg of LUPF and ds-LUPF were dissolved in 1 mL of D_2_O. One- and two-dimensional NMR spectra were recorded using an NMR spectrometer (Bruker BioSpin GmbH, Rheinstetten, Germany).

#### 2.3.7. Methylation Analysis

Methylation analysis of LUPF and ds-LUPF was performed according to a previously reported method [24]. Briefly, samples (20 mg) were methylated using NaOH-DMSO with methyl iodide under an N_2_ atmosphere. The methylated products were hydrolyzed with formic acid followed by TFA, reduced with NaBH_4_, and acetylated. The resulting partially methylated alditol acetates were analyzed by GC-MS (Agilent Technologies, Inc., Santa Clara, CA, USA) [25].

### 2.4. Preparation of S. typhimurium (ST) Suspension

ST was cultured in Luria–Bertani broth at 37 °C, harvested by centrifugation (4000× *g*, 4 °C for 10 min), washed with PBS, and resuspended in PBS to 1 × 10^7^ CFU/mL [26].

### 2.5. Mice Experiment

Specific pathogen-free male BALB/c mice (6 weeks old, 20–23 g) were obtained from Changsheng Biotechnology Co., Ltd. (Benxi, China). Mice were housed in an IVC system under standard conditions (12 h light/dark cycle, 23–26 °C, relative humidity of 45–55%). Mice had ad libitum access to water and food throughout the experiment. Mice-related procedures followed the National Institutes of Health guidelines for the Care and Use of Laboratory Animals and were approved by the Animal Experiment Ethics Committee of Dalian Polytechnic University (Approval No. DLPU2024DT003).

Mice were randomly divided into four groups: control (CN), ST infection model (ST), ST + UPF treatment (UPF), and ST + LUPF treatment (LUPF) groups, n = 7/group. To disrupt the gut microbiota, the ST, UPF, and LUPF groups were orally administered 200 μL antibiotic mixture (gentamicin sulfate (23 mg/kg), cefradine (23 mg/kg), and metronidazole (30 mg/kg)) for 10 days. Subsequently, these mice received daily oral gavage of 200 μL ST suspension for 13 days. During the infection period, mice in the UPF and LUPF groups were additionally treated with 200 mg/kg/day of UPF or LUPF, respectively [11], while the ST and CN groups received an equivalent volume of PBS. Mice feces were collected at the experimental endpoint for microbiota sequencing analysis. Finally, mice were anesthetized with isoflurane, and blood were collected from the retro-orbital sinus. After euthanasia by cervical dislocation, the liver, colon, and spleen were excised and measured. Organ indices were calculated according to a previously reported method [27].

### 2.6. Quantification of S. typhimurium in Organ Tissues

The liver (~0.5 g), spleen (~0.5 g), and proximal colon (~1 cm) were homogenized in PBS (1:5; *w*/*v*) using a glass homogenizer. Serial dilutions of the homogenates were plated onto xylose lysine deoxycholate (XLD) agar plates, and ST colonies were counted after incubation at 37 °C for 24 h.

### 2.7. Histological Analysis

Colon sections were prepared and stained with hematoxylin and eosin (H&E) for histological analysis under a light microscope (Leica Microsystems GmbH, Wetzlar, Germany). Histopathological scoring was performed according to the previously established criteria: 0, no inflammation or crypt damage; 1, mild inflammation with minimal crypt loss; 2, moderate inflammation with focal crypt damage; and 3, severe inflammation with extensive crypt destruction and inflammatory cell infiltration [28]. Periodic acid–Schiff (PAS) staining was conducted to evaluate mucin content, following a previously established protocol [29]. Quantitative analysis of PAS-stained sections was performed by measuring the PAS-positive area using ImageJ software (version 1.53, NIH, Bethesda, MD, USA) in a double-blinded manner.

For immunohistochemistry (IHC), tissue sections were dewaxed, rehydrated, treated with 3% H_2_O_2_ and 10% goat serum. The sections were incubated overnight at 4 °C with primary antibodies against MUC2, ZO-1, claudin, and occludin (1:200), followed by incubation with HRP-conjugated secondary antibody (1:1000). The sections were stained with diaminobenzidine and hematoxylin. Images were acquired using a light microscope (Leica, Germany) and analyzed in a double-blind manner according to a previously reported method [30].

### 2.8. Measurement of Biochemical Parameters

Colon tissues were homogenized in ice-cold PBS (1:9; *w*/*v*). After centrifugation (3000× *g*, 10 min, 4 °C), the levels of myeloperoxidase (MPO, A044-1-1), malondialdehyde (MDA, A003-1-2), catalase (CAT, A007-1-1), total superoxide dismutase (T-SOD, A001-1-2), and nitric oxide (NO, A013-2-1) were determined using kits (Jiancheng Bioengineering Institute, Nanjing, China). Levels of lipopolysaccharides (LPS, ml037221), tumor necrosis factor-α (TNF-α, ml002095), interleukin-1β (IL-1β, ml098416), and IL-10 (ml037873) were quantified using ELISA kits (Enzyme-linked Biotechnology Co., Ltd., Shanghai, China).

### 2.9. Measurement of Short-Chain Fatty Acids (SCFAs)

Fecal SCFAs were analyzed using GC (Shimadzu, Corporation, Kyoto, Japan) according to our previously established protocol [31]. Briefly, 50 mg of fecal sample was homogenized in diethyl ether containing 2-ethylbutyrate (1%, *v*/*v*) and acidified with 50% H_2_SO_4_. After centrifugation (12,000× *g*, 10 min), the supernatant was dried over anhydrous Na_2_SO_4_ and filtered via a 0.22 μm membrane before analysis. Chromatographic separation was performed using a GC-FID system equipped with a DB-FFAP column. A flame ionization detector (FID) was operated at 230 °C. Nitrogen was used as the carrier gas at a flow rate of 1.0 mL/min. The injection volume was 1 μL with a split ratio of 1:10. The oven temperature program was as follows: initial temperature was maintained at 100 °C, increased to 180 °C at a rate of 5 °C/min, and then held at 180 °C for 4 min. SCFAs were identified by comparing retention times with authentic standards and quantified using external calibration curves.

### 2.10. Sequencing Analysis of the Fecal Microbiota

Fecal microbiota composition was analyzed by Biomarker Technology Co., Ltd. (Beijing, China) according to a previously described method [32]. Microbiota richness and diversity were evaluated using the ACE, Chao1, Simpson, and Shannon indices. Principal coordinate analysis (PCoA) was conducted to analyze similarities in microbial community structure among groups. Key bacterial taxa distinguishing different groups were further determined using linear discriminant analysis (LDA).

### 2.11. Metabolomic Analysis of Serum Samples

Serum sample preparation, HPLC/MS analysis, metabolite identification, and data processing were conducted according to our previously established method [33]. Briefly, serum samples were extracted using acetonitrile/methanol/water (2:2:1, *v*/*v*/*v*) containing internal standards and incubated at –30 °C for 4 h. After centrifugation (13,000× *g*, 4 °C, 15 min) and filtration through a 0.22 μm membrane, the extracts were separated on an XBridge BEH amide column (4.6 mm × 100 mm, 3.5 μm) using 20 mM ammonium acetate and acetonitrile as the mobile phase at 0.35 mL/min. Metabolites were detected using a TripleTOF 5600+ mass spectrometer operating in negative ion mode. Quality control (QC) samples prepared by mixing equal aliquots of all serum samples were injected after every 7 analytical runs. The coefficients of variation for retention time and peak intensity of QC samples were below 15%, confirming the stability and reproducibility of LC-MS platform. Data were processed with MS-DIAL, and metabolites with |log_2_ fold change| > 1 and *p* < 0.05 were considered significantly altered. Principal component analysis (PCA) and orthogonal partial least squares discriminant analysis (OPLS-DA) were performed to evaluate differences in metabolite profiles among groups. A heatmap was generated to visualize metabolites showing marked alterations between groups. Metabolic pathway enrichment analysis was conducted using the KEGG database.

### 2.12. Effect of LUPF on ST-Induced Inflammation In Vitro

#### 2.12.1. Cell Culture

RAW264.7 cells were cultured in DMEM at 37 °C in a humidified atmosphere containing 5% CO_2_. Cells were seeded into 96-well plates (1 × 10^4^ cells/well) and incubated for 24 h. After removal of the medium, cells were treated with ST (1 × 10^5^ CFU/well), UPF (5–800 μg/mL), LUPF (5–800 μg/mL), ST + UPF, or ST + LUPF at the corresponding concentrations, followed by an additional 24 h incubation [34]. Cell viability was evaluated using the MTT assay [35].

#### 2.12.2. Immunofluorescence Staining

Immunofluorescence staining was performed to assess the effect of LUPF on macrophage polarization, with CD86 and CD163 as markers for M1 and M2 macrophages, respectively, according to a previously reported method [36]. Cells were fixed with 4% paraformaldehyde, permeabilized with 0.1% Triton X-100, and incubated with primary antibodies, followed by Alexa Fluor-conjugated secondary antibodies (1:500). Fluorescence images were acquired using a fluorescence microscope, and fluorescence intensity was quantified using ImageJ software (Bethesda, MD, USA).

#### 2.12.3. Quantitative Real-Time PCR

Total RNA was extracted from cells using TRIzol reagent, and cDNA was synthesized using a cDNA synthesis kit (RR047A, Takara Bio Inc., Kusatsu, Takara). The mRNA expression levels of genes were quantified by RT-PCR with the primers listed in Table S1. Relative gene expression was calculated using the 2^−∆∆Ct^ method, with *β-actin* as the reference gene [37].

### 2.13. Western Blotting

The expression levels of proteins related to the NF-κB and MAPK pathways, as well as Nrf2, in colon tissues were analyzed by Western blotting as previously described [38]. Briefly, proteins were separated by SDS–PAGE and transferred to PVDF membranes. The membranes were then incubated with primary antibodies (1:1000), followed by HRP-conjugated secondary antibodies (1:5000). Protein bands were visualized using an enhanced chemiluminescence system and quantified with ImageJ software.

### 2.14. Statistical Analysis

All data are expressed as mean ± standard deviation (SD). A one-way ANOVA followed by Tukey’s multiple-comparisons test was used to analyze statistical differences among groups in GraphPad Prism 8.0 software. A *p* value < 0.05 was considered statistically significant (* *p* < 0.05, ** *p* < 0.01, and *** *p* < 0.001, **** *p* < 0.0001).

## 3. Results and Discussion

### 3.1. Structural Characterization of LUPF

#### 3.1.1. General Structural Features of LUPF

**Figure 1:**
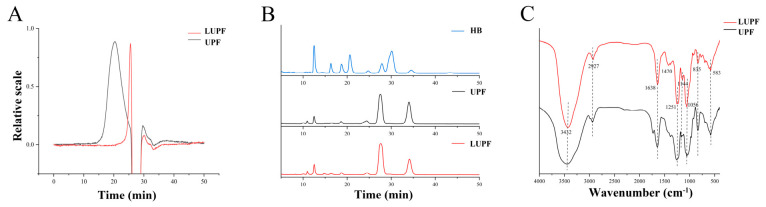
The characterization of LUPF. Mw of LUPF (**A**). Monosaccharide composition of LUPF (**B**). FT-IR spectra (**C**).

The Mw of LUPF was determined to be approximately 4.2 kDa, and its composition consisted of neutral sugars (64.3 ± 1.6%), sulfate groups (18.6 ± 1.3%), and uronic acids (2.5 ± 0.6%). LUPF exhibited a single and symmetrical peak in the HPGPC profile (Figure 1A), indicating good homogeneity. Monosaccharide composition analysis revealed that LUPF was primarily composed of Gal and Fuc, with a mass ratio of 9.9:4.5, along with a smaller amount of glucuronic acid (GlcA) (Figure 1B). Compared with UPF, no monosaccharides were completely absent in LUPF; however, distinct changes in monosaccharide proportions were observed after depolymerization. In particular, the Gal/Fuc ratio increased from 1.35 in UPF to 2.20 in LUPF. These changes could be attributed to the different susceptibilities of main- and branch-chain glycosidic linkages to acid hydrolysis during the depolymerization process.

#### 3.1.2. FT-IR Analysis

The FT-IR spectrum of LUPF exhibited the expected characteristic absorption bands of sulfated polysaccharide, confirming the preservation of its main structural features after depolymerization. As shown in Figure 1C, the peak at 3432 cm^−1^ corresponds to O-H stretching vibrations, while the peak at 2927 cm^−1^ is attributed to C-H stretching [39]. The absorption peak at 1638 cm^−1^ reflects the bending vibration and asymmetric stretching of C=O groups [40], indicating the presence of uronic acids. The stretching vibrations of C-O-C bonds are evidenced by the absorption peak at 1056 cm^−1^ [41]. The signals at 1251 cm^−1^ and 835 cm^−1^ correspond to asymmetric S=O stretching and C-O-S vibrations in the axial position, respectively. The absorption peak at 583 cm^−1^, assigned to S=O stretching vibrations, further confirmed the presence of sulfate groups in LUPF [42].

#### 3.1.3. Methylation and NMR Analysis

Methylation analysis showed that, after desulfation, the relative proportions of the linear residues → 4)-Fuc*p*-(1→ and →3)-Fuc*p*-(1 → increased slightly, whereas the branch point residue → 3,4)-Fuc*p*-(1 → decreased. Because desulfation was incomplete under the mild reaction conditions and the observed changes were relatively small, these results should be considered as preliminary observations. The relatively high levels of →6)-Gal*p*-(1→ and →3)-Gal*p*-(1 → in both LUPF and ds-LUPF suggest that these linkages may form the main polysaccharide backbone. NMR analysis (Figure S1) tentatively identified six anomeric proton signals. Comparison of the spectra of LUPF and ds-LUPF revealed slight upfield shifts for several fucose residues after desulfation, which is consistent with partial removal of sulfate groups from the C3 and/or C4 positions. Taken together, the methylation and NMR data suggest that LUPF is a sulfated fucoidan potentially containing a backbone mainly composed of →6)-Gal*p*-(1→ and →3)-Gal*p*-(1 → linkages, with some fucose residues substituted by sulfate groups at C3 or C4 [43]. Detailed data are provided in Tables S2 and S3.

### 3.2. LUPF Alleviated ST-Induced Multi-Organ Damage

**Figure 2:**
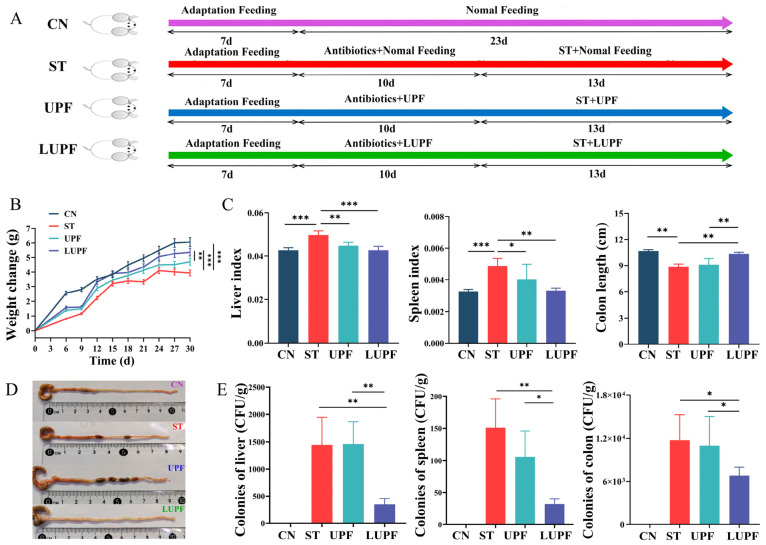
Effects of LUPF on body weight, organ indices, and bacterial translocation in ST-infected mice. Flowchart of the mouse experiment (**A**). Body weight changes (**B**). Liver index, spleen index, and colon length (**C**). Representative images of colon morphology (**D**). Quantification of ST colonies in the liver, spleen, and colon (**E**). Data are expressed as mean ± SD (n = 7) and analyzed by a one-way ANOVA, followed by Tukey’s test. * *p* < 0.05, ** *p* < 0.01, and *** *p* < 0.001.

The animal experiment procedure is depicted in Figure 2A. ST infection caused marked body weight loss, increased liver and spleen indices, and shortened colon length, indicating severe systemic and intestinal injury (Figure 2B–D). Treatment with UPF and LUPF alleviated ST-induced weight loss and partially normalized liver and spleen indices. Notably, LUPF significantly restored colon length, whereas no marked improvement was observed in the UPF group, suggesting a stronger protective effect of LUPF on intestinal injury. Quantification analysis of the bacterial load revealed markedly elevated ST colonization in the liver, spleen, and colon of ST-infected mice (Figure 2E), confirming systemic translocation of ST. Although UPF did not markedly reduce ST burden in these organs (*p* > 0.05), LUPF significantly decreased ST counts, indicating enhanced antimicrobial and/or barrier-protective activity. These findings suggest that LUPF provides more effective protection against ST-induced organ damage than UPF. Consistent with our findings, previous studies have demonstrated that certain oligosaccharides exert protective effects against ST infection in mice. For example, xylooligosaccharides (XOS) mitigated ST-induced body weight loss, colon shortening, and increased liver and spleen indices in mice [44]. Similarly, galactooligosaccharides promoted ST clearance in infected chickens, potentially through gut microbiota modulation independent of direct immune responses [45]. These results support the potential of LUPF as a promising prebiotic agent against ST-associated disorders.

### 3.3. LUPF Improved ST-Induced Serum Metabolic Abnormalities

**Figure 3:**
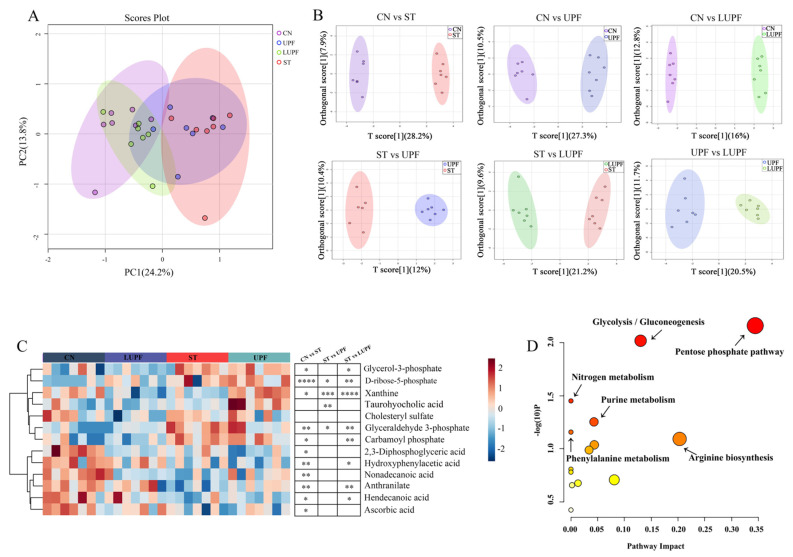
LUPF ameliorated ST-induced serum metabolic disturbances. PCA (**A**) and OPLS-DA of serum metabolites among groups (**B**). Heatmap of significantly altered metabolites (**C**). KEGG pathway enrichment analysis of differential metabolites (**D**). Data are analyzed using Student’s *t*-test. n = 7. * *p* < 0.05, ** *p* < 0.01, *** *p* < 0.001, and **** *p* < 0.0001.

ST infection induces not only local intestinal injury but also systemic metabolic disturbances. Metabolomic profiling provides valuable insights into disease pathogenesis and facilitates the identification of potential predictive biomarkers [46]. Given the limitations of current infection-related biomarkers, increasing attention has been directed toward discovering alternative indicators through metabolomics approaches [47]. In this study, serum metabolomics analysis was conducted based on HPLC–MS, and representative chromatograms are shown in Figure S2. PCA showed that the two principal components explained 37.2% of the total variance, with PC1 and PC2 accounting for 24.2% and 13.8%, respectively (Figure 3A). A clear separation between the ST and CN groups was observed, indicating substantial metabolic disturbances induced by ST infection. Notably, the metabolic profiles of the LUPF group showed considerable overlap with that of the CN group, suggesting partial restoration of metabolic homeostasis, whereas the UPF group remained closer to the ST group. OPLS-DA further confirmed distinct separations among all groups (Figure 3B), indicating that both LUPF and UPF modulated ST-induced metabolic alterations, with LUPF exerting a stronger regulatory effect. LPS, a major component of the outer membrane of Gram-negative bacteria such as ST, is known to induce systemic inflammation and metabolic dysfunction. Previous studies have shown that glycyrrhiza polysaccharides restored intestinal barrier function and improved serum metabolic profiles in LPS-treated mice [48]. These results suggest that LUPF may alleviate ST-induced systemic injury through modulation of serum metabolic homeostasis.

Heatmap analysis of significantly altered metabolites revealed that ST infection increased the levels of glycerol-3-phosphate, cholesteryl sulfate, D-ribose-5-phosphate, glyceraldehyde-3-phosphate, and carbamoyl phosphate (Figure 3C). In contrast, hydroxyphenylacetic acid, 2,3-diphosphoglyceric acid, anthranilate, hendecanoic acid, nonadecanoic acid, and ascorbic acid were markedly decreased. LUPF treatment reduced the levels of carbamoyl phosphate, glycerol-3-phosphate, cholesteryl sulfate, and glyceraldehyde-3-phosphate, while elevating hydroxyphenylacetic acid, anthranilate, ascorbic acid, and hendecanoic acid. In contrast, UPF exerted relatively weaker regulatory effects on these metabolites. KEGG pathway enrichment analysis demonstrated that these differential metabolites are mainly involved in several metabolic pathways, including the pentose phosphate pathway, nitrogen metabolism, phenylalanine metabolism, glycolysis/gluconeogenesis, purine metabolism, and arginine biosynthesis (Figure 3D). Similar metabolic pathways have also been implicated in the alleviation of ST-associated metabolic disturbances by *Bifidobacterium animals* [49]. Glycerol-3-phosphate, an intermediate of glycolysis, has been reported to promote M1 macrophage polarization and pro-inflammatory cytokine production [50]. In contrast, mannose reduced glycerol-3-phosphate level and suppressed TNF-α secretion, thereby restoring intestinal immune metabolic balance [51]. In addition, the pentose phosphate pathway plays an important role in regulating redox balance and inflammatory responses [52]. Pectic polysaccharide from *Aconitum* roots alleviated colonic inflammation via modulation of this pathway [53]. Furthermore, amino acid metabolism is closely associated with immune regulation and microbial homeostasis, and balanced amino acid diets have been shown to improve gut microbiota composition and alleviate intestinal inflammation [54]. These results suggest that LUPF alleviates ST-induced systemic metabolic disturbances, thereby contributing to its protective effects.

### 3.4. LUPF Protected Intestinal Barrier Integrity in ST-Treated Mice

**Figure 4:**
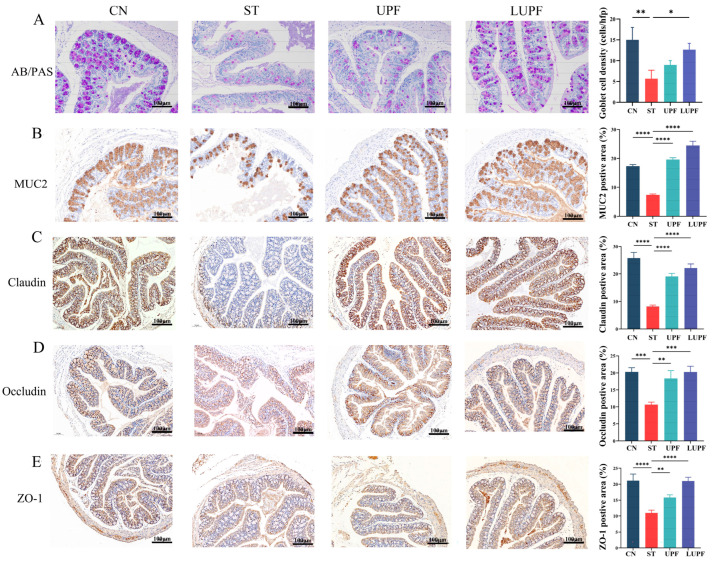
LUPF protected against ST-induced colonic injury. AB/PAS staining of colon tissues (**A**). IHC staining and quantitative analysis for MUC2 (**B**), claudin (**C**), occludin (**D**), and ZO-1 (**E**) expression in colon tissues. Data are expressed as mean ± SD (n = 3). Statistical analysis was performed using a one-way ANOVA, followed by Tukey’s test. * *p* < 0.05, ** *p* < 0.01, *** *p* < 0.001, and **** *p* < 0.0001.

The GI tract serves as the primary interface between the host and the external environment, and represents the main entry route for ST infection. The intestinal barrier is a multilayered defense system comprising biological, chemical, and epithelial barriers, which protect the host against enteric pathogens [55]. Disruption of this barrier is a hallmark of ST-induced intestinal injury. MUC2 plays a critical role in maintaining the mucosal layer and regulating intestinal permeability. Reduced MUC2 expression is closely associated with increased intestinal permeability and enhanced susceptibility to infection [56]. PAS staining demonstrated that ST infection markedly reduced goblet cell density and disrupted colonic crypt architecture (Figure 4A). Consistently, IHC staining revealed a substantial decrease in MUC2 expression in the colonic tissues of ST-infected mice, indicating severe impairment of the mucosal barrier (Figure 4B). Both LUPF and UPF treatment preserved goblet cell density, maintained crypt structure, and enhanced MUC2 secretion, with LUPF exhibiting a more pronounced protective effect. Tight junction proteins, including occludin, claudin, and ZO family proteins, form the most apical junctional complex and are essential for maintaining paracellular permeability and epithelial integrity [57]. ST infection significantly reduced the expression of claudin, occludin, and ZO-1 in colonic tissues (Figure 4C–E). LUPF treatment markedly upregulated the expression of these proteins. No significant differences were observed between the LUPF and UPF groups. This is consistent with previous studies showing that inulin alleviated ST-induced intestinal injury by increasing MUC2 and claudin-1 expression in chickens [58]. Similarly, galactooligosaccharides enhanced the expression of ZO-1 and claudin, thereby strengthening intestinal barrier function against *E. coli* infection [59]. These findings suggest that LUPF mitigates ST-induced intestinal injury by preserving intestinal barrier integrity.

### 3.5. LUPF Alleviated ST Infection-Induced Inflammation and Oxidative Stress

**Figure 5:**
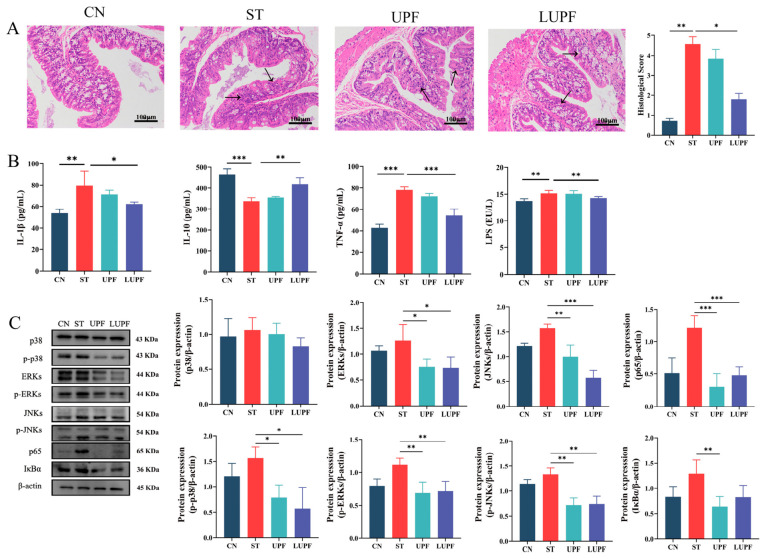
LUPF inhibited ST-induced inflammation in colon tissues. H&E staining and histopathological analysis of colon tissues (n = 3). Bar scale = 100 μm. Black arrows indicate inflammatory infiltration (**A**). Levels of IL-1β, IL-10, TNF-α, and LPS in colon tissues (n = 5) (**B**). Western blot analysis and quantitative evaluation of NF-κB and MAPK-related proteins, including p38, p-p38, ERKs, p-ERKs, JNKs, p-JNKs, p65, and IκBα (n = 3) (**C**). Data are expressed as mean ± SD. Statistical analysis was performed using a one-way ANOVA, followed by Tukey’s test. * *p* < 0.05, ** *p* < 0.01, and *** *p* < 0.001.

Histological analysis revealed that ST infection caused severe colonic tissue injury, characterized by inflammatory infiltration and crypt damage, resulting in significantly elevated histopathological scores (Figure 5A). In addition, ST infection markedly upregulated the levels of pro-inflammatory cytokine (IL-1β and TNF-α), while reducing anti-inflammatory IL-10 in colon tissues (Figure 5B). ST infection also markedly increased LPS levels. LUPF treatment markedly reduced IL-1β, TNF-α, and LPS levels while restoring IL-10 levels. In contrast, UPF showed no statistically significant effects on these inflammatory factors (*p* > 0.05). This is consistent with the fact that XOS mitigated ST-induced intestinal inflammation and mucosal injury in mice [44].

To further investigate the mechanisms underlying the anti-inflammatory effects of LUPF, the activation of the NF-κB and MAPK pathways was evaluated (Figure 5C). ST infection increased the expression of p65, p-ERK, JNKs, p-p38, p-JNKs, and IκBα, suggesting activation of the NF-κB and MAPK pathways. Although some changes did not reach statistical significance, the overall trend supported pathway activation in response to ST infection. UPF and LUPF significantly downregulated the expression levels of ERKs, JNKs, p65, p-p38, p-ERKs, and p-JNKs, with no significant differences observed between them. This indicates that UPF and LUPF suppress the activation of NF-κB and MAPK signaling pathways, which are known to regulate inflammatory responses through modulation of pro-inflammatory cytokines, chemokines, and adhesion molecules [60]. This is consistent with previous studies that ST-induced inflammation is closely associated with activation of NF-κB and MAPK pathways [61]. For example, konjac glucomannan alleviated colitis in ST-infected mice through suppression of NF-κB signaling [26], while exopolysaccharides from *Lactobacillus rhamnosus* inhibited MAPK and NF-κB activation to exert anti-inflammatory effects [62]. These results suggest that LUPF alleviates ST-induced inflammation, at least in part, through suppression of NF-κB and MAPK pathways.

**Figure 6:**
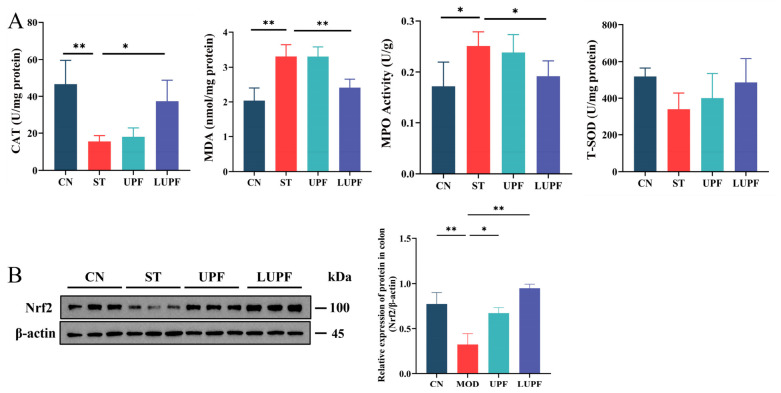
LUPF alleviated oxidative stress in colon tissues. Levels of CAT, MDA, MPO, and T-SOD, n = 5 (**A**). Representative Western blot images and quantitative analysis of Nrf2 protein, n = 3 (**B**). Data are expressed as mean ± SD. Statistical analysis was performed using a one-way ANOVA followed by Tukey’s test. * *p* < 0.05, and ** *p* < 0.01.

In addition to inflammation, oxidative stress also plays a critical role in ST-induced intestinal injury. ST-infected mice exhibited markedly decreased CAT levels and increased MDA and MPO levels in the colon tissues, whereas no significant difference was observed in T-SOD levels (Figure 6A). LUPF significantly increased CAT levels and reduced MDA and MPO levels, while UPF showed no marked effects on these oxidative stress-related markers. The Nrf2 pathway is a key cellular defense mechanism against oxidative stress, mainly through regulation of CAT, GSH, and HO-1 [63]. In this study, ST infection significantly reduced Nrf2 expression, whereas LUPF and UPF restored its levels (Figure 6B). This indicates that LUPF alleviates ST-induced oxidative stress through activation of the Nrf2 pathway. Similarly, *L. rhamnosus* exopolysaccharides mitigated ST-induced colonic injury by reducing MDA levels and increasing CAT activity [62]. These results suggest that LUPF exerts protective effects against ST-induced intestinal injury through regulation of inflammatory responses and oxidative stress pathways.

### 3.6. LUPF Modulated ST Infection-Induced Gut Microbiota Dysbiosis

**Figure 7:**
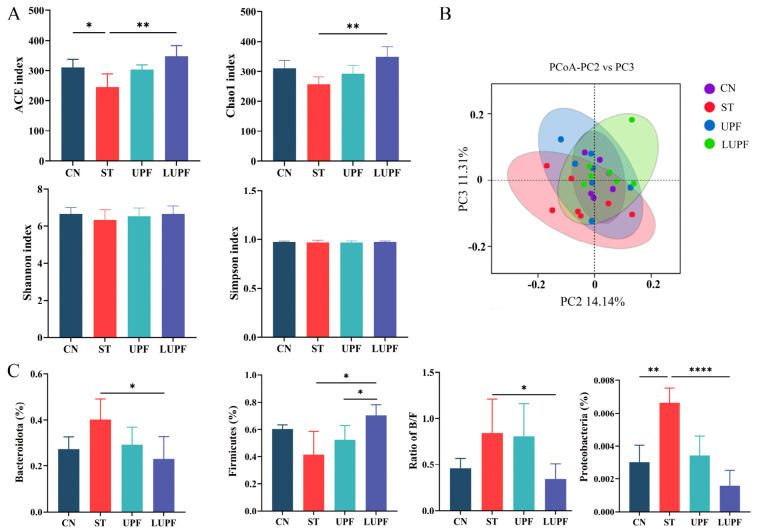
LUPF modulated ST-induced dysbiosis of gut microbiota. Ace, Chao1, Shannon, and Simpson indices (**A**). PCoA (**B**). The levels of Bacteroidota, Firmicutes, Proteobacteria, and the ratio of Bacteroidetes to Firmicutes (B/F) (**C**). Data are expressed as mean ± SD (n = 5). Statistical analysis was performed using one-way ANOVA, followed by Tukey’s test. * *p* < 0.05, ** *p* < 0.01, and **** *p* < 0.0001.

The gut microbiota plays a vital role in host defense against pathogen-induced diseases through mechanisms including immune modulation and competitive exclusion of intestinal niches [5]. As shown in Figure 7A, the ACE and Chao1 indices were decreased in the ST group, although no statistically differences were observed (*p* > 0.05). No obvious differences were found in the Shannon and Simpson indices. UPF exerted little effect on these indices, whereas LUPF increased the ACE and Chao1 indices, suggesting a partial restoration of microbial richness. PCoA revealed no obvious separation among groups (Figure 7B), suggesting that ST infection did not induce dramatic alterations in the overall community structure. ST infection significantly elevated Bacteroidota and Proteobacteria (*p* < 0.05) levels while reducing Firmicutes levels, resulting in an elevated Bacteroidota/Firmicutes (B/F) ratio (Figure 7C). LUPF reversed these alterations by decreasing Bacteroidota and Proteobacteria levels and increasing Firmicutes levels, thereby reducing the B/F ratio. No significant differences were observed between the UPF and ST groups on these phyla. Recent studies showed that ST infection differentially affects Firmicutes and Bacteroidota levels while consistently increasing Proteobacteria levels [64,65,66]. Clinical evidence further demonstrated that patients infected with enteric pathogens such as *Salmonella* and *Shigella*, exhibited elevated Proteobacteria levels, whereas healthy individuals generally showed higher levels of Bacteroidota and Firmicutes [67]. The B/F ratio is widely regarded as an indicator of gut dysbiosis associated with altered energy metabolism and impaired intestinal barrier function [68]. In addition, increased Proteobacteria is considered a microbial signature of intestinal and systemic diseases [69]. These findings suggest that LUPF alleviates ST-induced gut dysbiosis, particularly through suppression of Proteobacteria.

**Figure 8:**
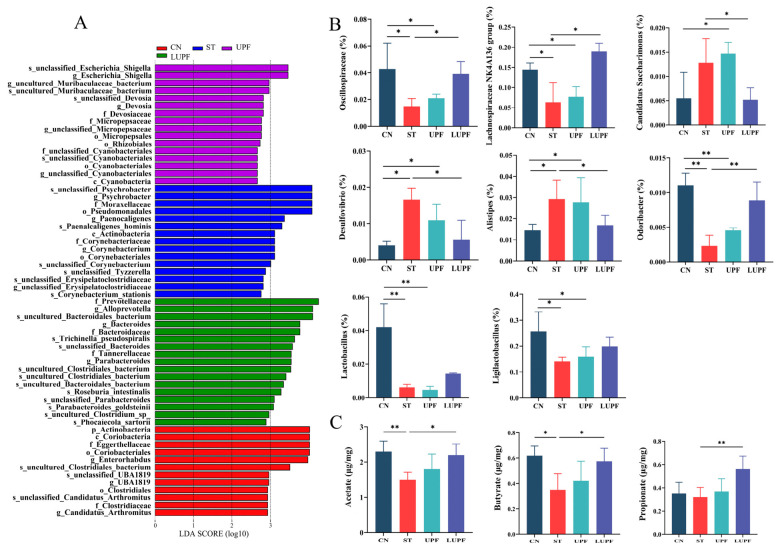
Effect of LUPF on specific gut microbes and SCFA production. LDA (**A**). Comparative analysis of specific bacteria at the genus level (**B**). Effect of LUPF on the production of acetate, butyrate, and propionate (**C**). Data are expressed as mean ± SD (n = 5). * *p* < 0.05 and ** *p* < 0.01.

The effects of ST infection, UPF, and LUPF on gut microbiota were further analyzed using LDA (Figure 8A). A total of 60 dominant bacterial taxa were identified among groups. Among them, 16 were enriched in the UPF group, 15 in the ST group, 17 in the LUPF group, and 12 in the CN group. ST infection increased the levels of potentially harmful taxa, including *Alistipes*, *Saccharimonas*, and *Desulfovibrio*, while reducing beneficial taxa such as Oscillospiraceae, Lachnospiraceae NK4A136 group, *Odoribacter*, *Lactobacillus*, and *Ligilactobacillus* (Figure 8B). UPF had limited effects on these taxa (*p* > 0.05), whereas LUPF reduced *Alistipes*, *Saccharimonas*, and *Desulfovibrio* while increasing the levels of Lachnospiraceae NK4A136 group, Oscillospiraceae, *Odoribacter*, and *Lactobacillus* (*p* > 0.05). Although some differences did not reach statistical significance, the overall trends suggested a beneficial modulatory effect of LUUPF on the gut microbiota. Several of these microbial alterations are functionally associated with inflammation and host health. *Saccharimonas* has been linked to inflammatory conditions, including gingivitis, periodontal dysfunction, and obesity [70,71]. *Desulfovibrio*, regarded as a potential intestinal pathogen, has been reported to aggravate colitis [72]. In contrast, the Lachnospiraceae NK4A136 group contributes to intestinal homeostasis through butyrate production [73]. *Oscillospiraceae* has been positively associated with certain health biomarkers such as SCFAs and negatively associated with inflammation [74]. In addition, *Odoribacter* may restrict the proximal spread of colitis and reduce mucosal inflammation severity [75]. These findings suggest that LUPF alleviates ST-induced intestinal inflammation by suppressing potentially pathogenic taxa while enriching beneficial taxa such as SCFA-producing bacteria.

### 3.7. LUPF Promoted the Production of Intestinal SCFAs

SCFAs, primarily produced through microbial fermentation of polysaccharides in the colon, play critical roles in maintaining intestinal and systemic health. SCFA production is influenced by gut microbiota composition as well as the structural characteristics of polysaccharides [76]. Previous studies have shown that ST infection disrupts gut microbial homeostasis, resulting in reduced SCFA production and altered SCFA profiles [77,78]. SCFAs exert protective effects through multiple mechanisms, including direct inhibition of enteric pathogens via protein acylation, enhancement of intestinal barrier integrity, and regulation of immune responses [79]. ST infection significantly reduced the levels of acetate and butyrate, whereas no significant differences were observed in propionate (Figure 8C). LUPF significantly increased acetate, propionate, and butyrate production, while no significant differences were observed between the ST and UPF groups. These results suggest that the Mw, structure, and type of polysaccharides may strongly influence their microbial fermentation efficiency and subsequent SCFA production [80]. The elevated production of SCFA observed with LUPF may be attributed to its low Mw that favor microbial fermentation. Previous study showed that inulin ameliorated ST-induced gut dysbiosis and restored SCFA production, thereby contributing to microbial and host homeostasis [77]. In addition, direct butyrate supplementation reduced ST colonization in chickens by modulating gut microbiota composition and strengthening intestinal barrier function [81]. These results suggest that LUPF alleviates ST-induced intestinal dysfunction through restoration of SCFA production, highlighting the importance of gut microbiota-SCFA-host interactions in its protective effects.

### 3.8. LUPF Alleviated Inflammation by Regulating Macrophage Polarization

**Figure 9:**
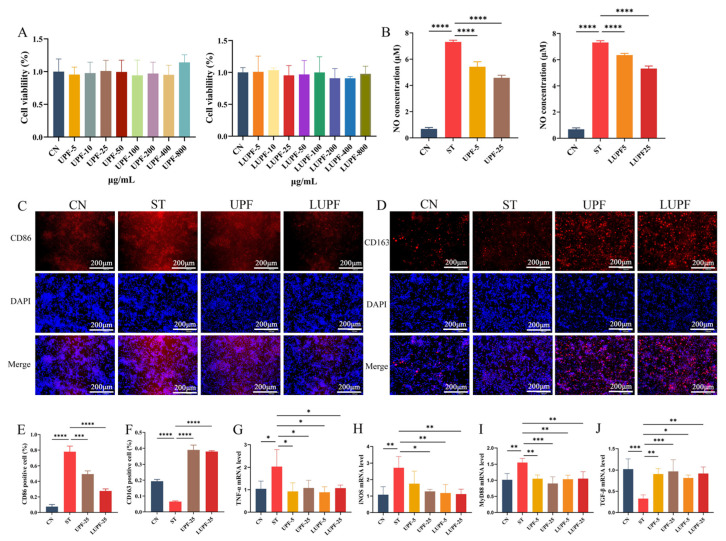
Effect of LUPF on ST-stimulated inflammation in RAW264.7 cells. Effect of UPF and LUPF on cell viability (**A**). NO level (**B**). Immunofluorescence staining and quantitative analysis of macrophage polarization based on markers CD86 and CD163 (**C**–**F**). The mRNA expression levels of *TNF-α*, *iNOS*, *MyD88*, and *TGF-β* (**G**–**J**). Data are expressed as mean ± SD (n = 3). * *p* < 0.05, ** *p* < 0.01, *** *p* < 0.001, and **** *p* < 0.0001.

To further investigate the direct protective effects of LUPF against ST-induced inflammation, an in vitro cell model was established. Cytotoxicity analysis demonstrated that neither LUPF nor UPF exhibited obvious toxicity at concentrations up to 800 μg/mL (Figure 9A). Based on these results, 5 and 25 μg/mL of UPF and LUPF were selected for anti-inflammatory evaluation. ST stimulation significantly increased NO production, whereas LUPF and UPF reduced NO levels in a concentration-dependent manner (Figure 9B). Higher concentrations of UPF and LUPF exhibited stronger inhibitory effects on NO production, although no statistically differences were observed between them (*p* > 0.05). Additionally, immunofluorescence analysis further demonstrated that ST stimulation increased CD86 expression and decreased CD163 expression, indicating that ST promoted macrophage polarization toward the pro-inflammatory M1 phenotype (Figure 9C–F). These changes were accompanied by increased expression of pro-inflammatory markers, including TNF-α, iNOS, and MyD88, together with reduced TGF-β expression (Figure 9G–J). Treatment with 25 μg/mL of LUPF markedly decreased M1 macrophage polarization while promoting M2 macrophage polarization. Correspondingly, LUPF reduced TNF-α, iNOS, and MyD88 expression and increased TGF-β expression, and no statistically significant differences were detected between UPF and LUPF at the tested concentrations.

These results are consistent with previous studies showing that alginate oligosaccharides attenuated LPS-induced inflammation in RAW264.7 macrophages through modulation of inflammatory cytokine profiles [82]. M1 macrophage promotes inflammatory responses through secretion of pro-inflammatory cytokines, whereas M2 macrophages contribute to immune regulation and tissue repair by producing anti-inflammatory factors [83]. Given their sensitivity to microbial signals, macrophages are considered pivotal regulators of intestinal inflammation. For instance, chitosan oligosaccharides protected intestinal barrier integrity by regulating macrophage polarization via the MyD88/NF-κB signaling pathway [84]. Notably, the anti-inflammatory and macrophage polarization-regulating effects of LUPF were comparable to those of UPF, suggesting that Mw reduction may not markedly influence the direct immunomodulatory activity of UPF. Similar results have been reported for *Astragalus* polysaccharides and their low-Mw oligosaccharides, which exhibited comparable anti-inflammatory effects in macrophage models [85]. These results suggest that the superior protective effects of LUPF may be closely associated with systemic regulation and gut microbiota-mediated mechanisms rather than direct cellular immunomodulatory activity alone.

## 4. Conclusions

This study demonstrated the protective effects of LUPF against ST infection-induced intestinal and systemic injury. Compared to UPF, LUPF more effectively reduced ST colonization, preserved intestinal barrier integrity, modulated gut microbiota composition, and promoted SCFA production, thereby alleviating intestinal inflammation and oxidative stress. In addition, LUPF markedly improved ST-induced serum metabolic disturbances. Although LUPF also directly regulated macrophage polarization in vitro, its effects were comparable to those of UPF, suggesting that the superior protective effects of LUPF are more likely associated with enhanced microbiota regulation and fermentation-related properties rather than direct cellular immunomodulatory activity. These results highlight the potential of LUPF as a promising prebiotic candidate for mitigating ST-associated intestinal diseases through regulation of intestinal microecology and immune responses. However, several limitations require further elucidation, including the identification of the specific microbial taxa and metabolites responsible for the protective effects of LUPF and the detailed mechanisms underlying the interactions among LUPF, gut microbiota, and host signaling pathways. Additionally, the causal role of gut microbiota in LUPF-mediated protection was not directly confirmed because fecal microbiota transplantation experiments were not performed. Future studies should further evaluate the efficacy and mechanisms of LUPF in intestinal health management.

## Data Availability

The original contributions presented in the study are included in the article/Supplementary Materials, further inquiries can be directed to the corresponding author.

## Supplementary Materials

The following supporting information can be downloaded at: https://www.mdpi.com/article/10.3390/foods15122135/s1, Table S1: Primer sequences used for RT-PCR experiments; Table S2: Results of methylation analysis of LFUC and ds-LFUC; Table S3: ^1^H and ^13^C NMR chemical shifts in LFUC and ds-LFUC in D_2_O; Figure S1: Structural characterization of LUPF. ^1^H NMR spectrum (A). ^13^C NMR spectrum (B). HSQC (C). COSY (D). TOCSY (E) and NOESY (F) of LUPF. HSQC (G) and COSY (H) spectra of ds-LUPF; Figure S2: Representative extracted ion chromatograms (EIC) of four differential serum metabolites in control (CN) and ST-infected mice. Cholesteryl sulfate (A), Carbamoyl phosphate (B), D-Glyceraldehyde 3-phosphate (C), Glycerol 3-phosphate (D).
