# Supplementary material for: Low-Molecular-Weight Fucoidan from Undaria pinnatifida Mitigates Salmonella-Induced Injury Through Gut Microbiota and Immune Regulation
Source: Foods. 2026 Jun 13;15(12):2135. doi: 10.3390/foods15122135 (PMC13298705; doi:10.3390/foods15122135)

Low-molecular-weight fucoidan from *Undaria pinnatifida* mitigates *Salmonella*-induced injury through gut microbiota and immune regulation

Table S1 Primer sequences used for RT-PCR experiments.

Genes	Forward primer	Reverse primer
TNF-α	GCCTCTTCTCATTCCTGCTTGTGG	GTGGTTTGTGAGTGTGAGGGTCTG
iNOS	TCAGGTAGAGGCCTGGAAAA	GCATACAGGCAAAGAGCACA
MyD88	ACTTGTTAGACCGTGAGGAT	CTCGGACTCCTGGTTCTG
TGF-β	GCAACATGTGGAACTCTACCAG	GTTGGTATCCAGGGCTCTCC
CD86	ATATGACCGTTGTGTGTTCTGGA	AGGGCCACAGTAACTGAAGCTGTAA
CD163	ACGGCTGGAGCATGAATGAA	ATGCTTTCCCCACCCATCAT

Table S2 Results of methylation analysis of LFUC and ds-LFUC.

Deduced linkage	Methylated derivative	Characteristic fragments (m/z)	Molar ratio	
			LFUC	ds-LFUC
→4)-Fuc*p*-(1→	2,3-Me2-Fuc*p*	57、71、85、99、103、118、142、173、203	0.2	0.4
→3)-Fuc*p*-(1→	2,4-Me2-Fuc*p*	57、71、85、99、101、113、160、173、234	2.4	2.7
→3,4)-Fuc*p*-(1→	2-Me1-Fuc*p*	57、71、85、99、113、161、173、274	3.0	2.8
→6)-Gal*p*-(1→	2,3,4-Me3-Gal*p*	57、71、85、99、113、129、159、171、233	4.1	4.0
→3)-Gal*p*-(1→	2,4,6-Me3-Gal*p*	57、71、85、99、101、118、129、161、173	3.6	3.6
Gal*p*-(1→	2,3,4,6-Me4-Gal*p*	57、71、85、99、113、127、161	1.0	1.0

Table S3 ^{1}H and ^{13}C NMR chemical shifts of LFUC and ds-LFUC in D_2O.

Sample	Glycosyl residues		1H/13C (ppm)					
			H1/C1	H2/C2	H3/C3	H4/C4	H5/C5	H6/C6
LFUC	A	→4)-α-L-Fuc*p*3*S*-(1→	5.39/99.56	3.86/69.01	4.66/80.18	3.79/76.34	4.19/68.14	1.24/15.40
	B	→3)-α-L-Fuc*p*4*S*-(1→	5.24/92.31	3.84/67.16	4.26/78.64	4.46/80.12	4.13/70.22	1.25/15.62
	C	→3,4)-α-L-Fuc*p*-(1→	5.03/98.16	3.94/69.61	4.22/77.60	3.83/74.63	4.03/69.25	1.28/15.42
	D	→3)-β-D-Gal*p*-(1→	4.55/103.36	3.56/70.82	3.84/82.67	4.13/72.73	3.92/73.55	3.69/61.02
	E	β-D-Gal*p*-(1→	4.64/104.28	3.66/71.68	3.83/70.21	3.96/69.06	3.62/71.36	3.86/61.02
	F	→6)-β-D-Gal*p*-(1→	4.49/103.49	3.53/72.21	3.74/71.83	4.37/66.83	3.72/75.08	3.83/66.66
ds-LFUC	A	→4)-α-L-Fuc*p*3*S*-(1→	5.42/98.56	3.86/69.01	4.36/80.15	3.79/76.20	4.19/67.82	1.23/15.33
	B	→3)-α-L-Fuc*p*-(1→	5.24/100.93	3.84/67.59	4.26/78.54	3.92/73.35	4.13/69.20	1.25/15.62
	C	→3,4)-α-L-Fuc*p*-(1→	5.02/98.50	3.94/68.91	4.22/77.72	3.83/74.07	4.03/71.64	1.28/15.36
	D	→3)-β-D-Gal*p*-(1→	4.55/103.30	3.56/70.81	3.84/83.08	4.13/72.75	3.92/73.54	3.69/61.02
	E	β-D-Gal*p*-(1→	4.64/104.39	3.66/72.03	3.83/70.62	3.96/69.06	3.62/71.36	3.86/61.02
	F	→6)-β-D-Gal*p*-(1→	4.49/101.52	3.43/72.73	3.71/71.83	4.37/66.97	3.72/76.31	3.83/66.67

Figure S1 Structural characterization of LUPF. ^{1}H NMR spectrum (A). ^{13}C NMR spectrum (B). HSQC (C). COSY (D). TOCSY (E) and NOESY (F) of LUPF. HSQC (G) and COSY (H) spectra of ds-LUPF.

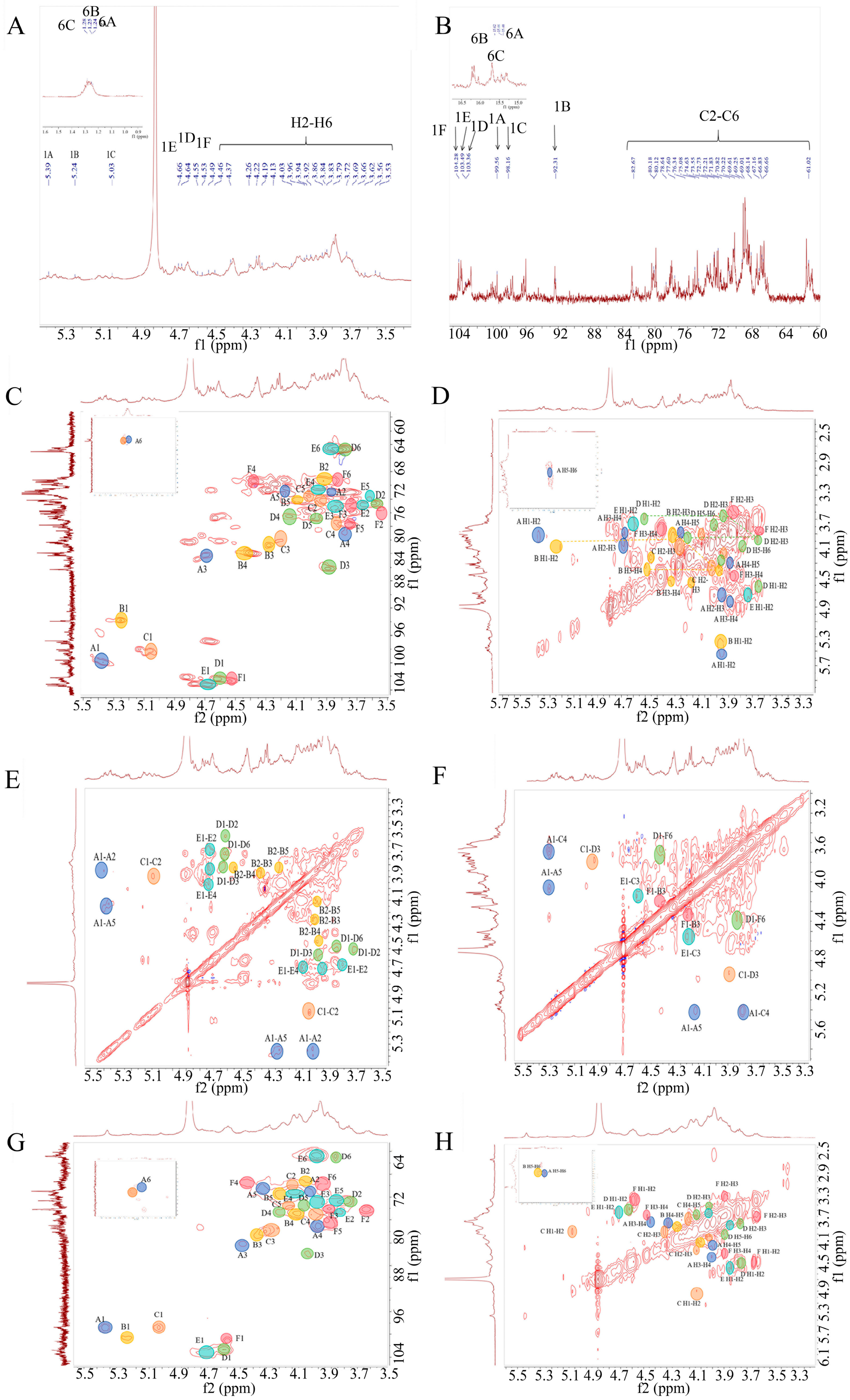

Figure S2 Representative extracted ion chromatograms (EIC) of four differential serum metabolites in control (CN) and ST-infected mice. Cholesteryl sulfate (A), Carbamoyl phosphate (B), D-Glyceraldehyde 3-phosphate (C), Glycerol 3-phosphate (D).

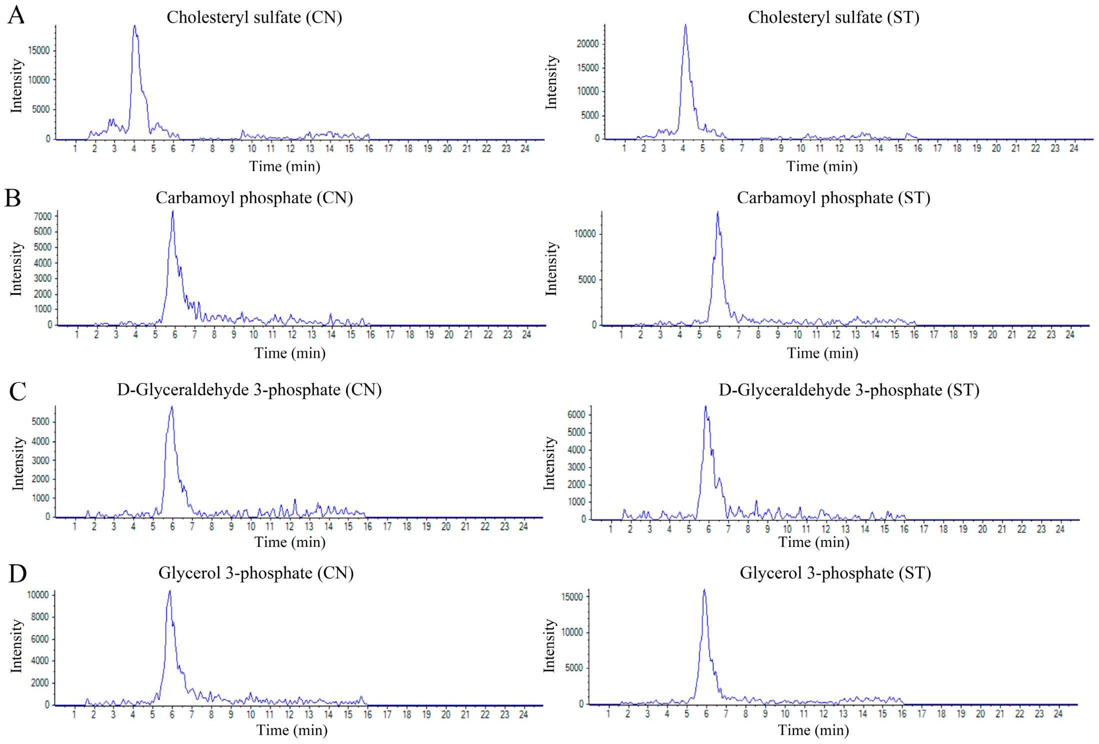

Supplement: Supplementary file 1 [file foods-15-02135-s001.zip › Supplementary data-0612.pdf]
